# A Process for Evaluating Quality Decision-Making Practices During the Development, Review and Reimbursement of Medicines

**DOI:** 10.34172/ijhpm.2020.86

**Published:** 2020-06-20

**Authors:** Magdalena Bujar, Neil McAuslane, Stuart Walker, Sam Salek

**Affiliations:** ^1^Department of Clinical and Pharmaceutical Sciences, School of Life & Medical Sciences, University of Hertfordshire, Hatfield, UK.; ^2^Centre for Innovation in Regulatory Science (CIRS), London, UK.

**Keywords:** Quality Decision-Making, QoDoS, Medicines Development, Regulatory Review, Health Technology Assessment

## Abstract

**Background:** The development of a medicine is not only underpinned by good science but also by Quality DecisionMaking Practices (QDMPs). Indeed, it is important to ensure that all organisations involved in the lifecycle of medicines are aligning their practices in decision-making to the QDMPs to ensure quality, transparent and consistent decisionmaking processes.

**Methods:** The aim of this study was to evaluate the practicality of QoDoS (Quality of Decision-Making Orientation Scheme) in assessing the incorporation of ten QDMPs during the development, review and reimbursement of medicines, illustrated by case studies with a pharmaceutical company, a regulatory authority and a health technology assessment (HTA) agency. Individuals from each organisation completed the 47-item QoDoS questionnaire.

**Results:** The results demonstrate the applicability of QoDoS in identifying favourable and unfavourable practices and in assessing the consistency and transparency of the QDMPs within each organisation, as well as across the different stakeholders. Furthermore, the study established the value of the methodology in raising awareness of the biases and best practices in decision-making, as well as having a basis for discussion for differences within and across stakeholders to promote consistency and alignment in decision-making. Finally, the QoDoS demonstrated the need for improvement across a number of decision-making practices for the 3 organisations such as the evaluation of alternatives and of the decision impact.

**Conclusion:** The QoDoS can be used to benchmark organisations’ decision-making practices to provide a basis for discussion to ultimately encourage a level of trust across and within organisations and helping to identify areas for improvement.

## Background

**Key Messages**

**Implications for policy makers**

- One way for organisations to determine how to improve their decision-making is to analyse the implementation of best practices in their decision-making frameworks; however, there is still a limited body of research regarding the evaluation of both the quality and transparency of the decision-making through which medicines become available.
- Previous research defined best practices in decision-making during the lifecycle of medicines, namely the ten Quality Decision-Making Practices (QDMPs) and described the development of an instrument, the Quality of Decision-Making Orientation Scheme (QoDoS) to measure their incorporation into individual and organisational processes.
- QoDoS can be applied as a diagnostic instrument within teams, committees, or departments in companies, regulatory authorities and health technology assessment (HTA) agencies for the routine assessment of quality of decision-making.
- This is the first assessment of QDMPs in companies and agencies, where the study determined favourable practices and those that might require improvement and identified common themes in quality decision-making across different organisations.
- This research further determined the applicability of the QoDoS tool in identifying favourable and unfavourable decision-making practices and in assessing the consistency and transparency of the QDMPs within companies and agencies.

**Implications for the public**

It is not always clear what explicit processes pharmaceutical companies, regulatory authorities and health technology assessment (HTA) agencies are using to make key strategic decisions. This publication describes a process for evaluating Quality Decision-Making Practices (QDMPs) during the development, review and reimbursement of medicines. Although, in general, the outcomes of the case studies were very positive in demonstrating alignment in terms of the incorporation of the QDMPs, they highlighted the need for improvement across a number of practices eg, need for better assignment of values and relative importance of decision criteria as well as the evaluation of alternatives and the need for impact analysis. Such greater transparency around the implementation of QDMPs would help to ensure trust by patients and public, letting them know that decision-making processes within these organisations are in line with best practice.

There has been increasing interest in research on decision-making over the past 2 decades and indeed the science of decision-making is well-established.^1-4^ It would therefore seem of value to explore whether the various findings and recommendations from this research have been incorporated into operations when decisions are made under conditions of uncertainty. Nevertheless, there is still a limited body of research regarding the evaluation of both the quality and transparency of decision-making through which medicines become available as well as how well the practices are aligned within and across organisations. Indeed, it is not always clear what explicit processes pharmaceutical companies, regulatory authorities and health technology assessment (HTA) agencies are using to make key strategic decisions. It is nevertheless important that there is better transparency around this, in order to ensure trust by patients and public, as well as across stakeholders in knowing that their decision-making processes are in line with best practice. This is particularly important with the growing trend and efforts to align regulatory and HTA standards and processes both within companies as well as across agencies, in order to further increase effectiveness and efficiency during the development, review and reimbursement of medicines.^5,6^

**Figure 1:**
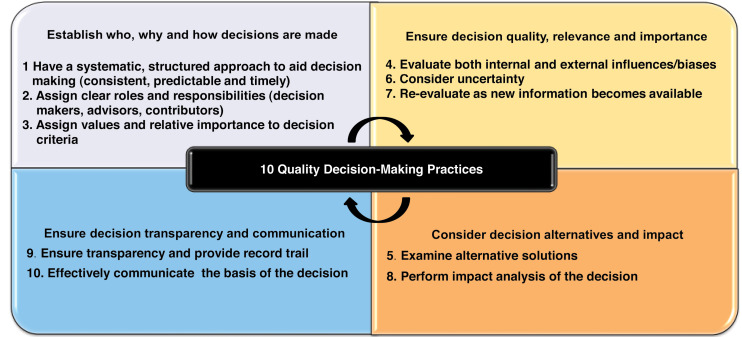
The 10 Quality Decision-Making Practices.

This gap in research into decision-making during medicines development was initially addressed through the research of Donelan and colleagues, which resulted in defining the best practices in decision-making during the lifecycle of medicines, namely the 10 Quality Decision-Making Practices (QDMPs) (Figure 1). Donelan et al also developed an instrument, the Quality of Decision-Making Orientation Scheme (QoDoS), which can be used to measure the incorporation of these 10 QDMPs into the operational processes of companies and agencies.^7,8^ The development of QoDoS has been underpinned by the well-established science of decision-making and it provides an assessment regarding its implementation into decision-making processes undertaken by individuals and organisations. Indeed, one way for organisations to determine how to improve their decision-making is first to analyse the implementation status of these best practices into their frameworks, which can be achieved with QoDoS. It should be noted however that QoDoS does not aim to assess the outcome but the focus is on the process through which decisions are made. Although a good process does not always guarantee a favourable outcome, the incorporation of best practices and having structured processes should increase the probability of more predictable, transparent and improved outcomes. In addition, it should ensure better trust and efficiency among teams and committees that are making such decisions, as well as across organisations particularly in the case of agency work-sharing or parallel HTA-regulatory approaches that are becoming increasingly more important.

A systematic review of the literature demonstrated QoDoS to be the most promising available technique for such assessments.^9^ Moreover, a pilot study with participants from pharmaceutical companies and regulatory authorities demonstrated the initial practicality of the QoDoS in assessing the strengths and weaknesses as well as similarities and differences in decision-making practices amongst individuals as well as their perception of their organisation.^10^

Furthermore, a recent study demonstrated the reliability and relevance of QoDoS in the decision-making of pharmaceutical companies, regulatory authorities and HTA agencies.^11^ These 2 properties are crucial to any future applications of the instrument, particularly in longitudinal studies, to ensure that potential changes in decision-making practices are a result of modified organisational processes rather than due to measurement error.^12^

 In order to determine the value of the methodology in assessing the level of implementation of the 10 QDMPs by the 3 types of organisations, case studies were initiated with participants from specific teams, committees, or departments across companies, regulatory and HTA agencies. This study was used to determine the factors that influence decision-making within organisations, including favourable practices and those that might require improvement, as well as to identify common themes in quality decision-making across different organisations to determine whether there is good alignment in the use of best practices in decision-making. The aim was therefore to evaluate the practicality of QoDoS in identifying the incorporation of the 10 QDMPs through illustrative case studies involving a pharmaceutical company, a regulatory authority and an HTA agency. The objectives were to:

- Evaluate the quality of the decision-making practices of the individuals and their perception of their respective organisation’s decision-making;
- Identify the favourable and unfavourable QDMPs;
- Assess the consistency of the QDMPs within each organisation including different decision-making groups as well as across organisations;
- Evaluate the feasibility and the perceived benefits of the study methodology based on initial feedback discussions as well as lessons learned.

## Methods

###  Design of the Study

This study was designed to examine the value of the QoDoS and the implementation of the 10 QDMPs in 3 case studies, each reflecting the decision-making environment of the respective stakeholders; that is, a pharmaceutical company, regulatory authority and HTA agency. The participants completed the QoDoS instrument (see Supplementary file 1) and each study was planned as a cross-sectional design, where the data were collected at one point in time. The purpose of each case study was to examine the organisation as a whole and to assess the QDMPs within a number of logical sub-units (eg, departments) within each organisation.

###  Assessment Technique

The 47-item QoDoS instrument (Supplementary file 1) consists of 2 parts: Part I relates to the organisation, comprising 2 domains (“Approach” and “Culture”) and Part II relates to the individual, grouped into 2 domains (“Competence” and “Style”), with each item to be rated on a 5-point Likert scale, where the following distinctions were made: Not at all = 0% of time; Sometimes = 25% of time; Frequently = 50% of time; Often = 75% of time; Always = 100% of time.

In addition, 4 demographic questions were used to collect data on gender, job title, professional experience and organisation type. The QoDoS items were rated as either favourable or unfavourable and based on this, the Likert scale response options were quantified by assigning scores. For QoDoS items considered as favourable practice, the following scores were assigned: Not at all = 0; Sometimes = 1; Frequently = 2; Often = 3; and Always = 4. For QoDoS items considered as unfavourable practice, the reverse scores were assigned: Not at all = 4; Sometimes = 3; Frequently = 2; Often = 1; and Always = 0 (Table 1).

**Table 1:** QoDoS Items Mapped to the 10 QDMPs

**10 QDMPs **	**QDMP Short Name**	**24 QoDoS Individual Items **	**23 QoDoSOrganisational Items **
1. Have a systematic, structured approach to aid decision-making (consistent, predictable and timely)	Structure	24, 25, 27, 30, 32, 35, 36, 39, 40, 43	3, 4, 11, 13, 14
2. Assign clear roles and responsibilities (decision-makers, advisors, contributors)	Roles	37	15, 23
3. Assign values and relative importance to decision criteria	Criteria	33, 34, 44	6, 7
4. Evaluate both internal and external influences/biases	Bias	38, 42	5, 17, 20, 21
5. Examine alternative solutions	Alternatives	28	8, 9
6. Consider uncertainty	Uncertainty	26, 45	10, 18
7. Re-evaluate as new information becomes available	New information	46	12, 19
8. Perform impact analysis of the decision	Impact	31, 47	1
9. Ensure transparency and provide a record trail	Transparency	29, 41	2, 16
10. Effectively communicate the basis of the decision	Communication		22

Abbreviations: QDMP, Quality Decision-Making Practice; QoDoS, Quality of Decision-Making Orientation Scheme. Underscored items indicate those that correspond to ‘unfavourable practice,’ whereas non-underscored items indicate those which represent ‘favourable practice.’

###  Study Participants

####  Selection Process and Inclusion Criteria

Using purposive sampling, 12 organisations were selected from across the participants in the Centre for Innovation in Regulatory Science research programmes.^13^ These include both companies (multinational companies with a global footprint) as well as regulatory and HTA agencies (mature and emerging agencies). Three companies, 3 regulatory and 3 HTA agencies were selected with the aim to identify one from each of the 3 different organisations. Positive responses were received from 2 pharmaceutical companies, 2 regulatory authorities and one HTA agency but only one of each organisation types were ultimately selected. This sampling process was considered appropriate as the aim was to produce illustrative case studies as opposed to generating company, regulatory authority, or HTA agency aggregated trends. The criteria for selecting the initial 12 organisations (first stage) and subsequently the 3 organisations for the case studies (second stage) are described below.

First stage inclusion criteria – The selected companies had research and development expenditures in excess of 1 billion USD,^14^ thereby reflecting their innovativeness. The 4 regulatory authorities were all classified as stringent regulatory authorities as defined by the World Health Organization (WHO).^15^ Lastly, to ensure that the organisations involved in these studies have well-established decision-making systems in place the 4 HTA agencies were selected based on size and maturity from those organisations that are part of the International Network of Agencies for Health Technology Assessment^16^ or European Network for Health Technology Assessment.^17^

 Second stage inclusion criteria – The 3 organisations were selected for the case studies based on availability of resources in terms of staff and time as well as on cohort size. This would enable calculation of the response variance as well as sub-group analysis on the dataset to identify differences and similarities and the consistency of implementation of the 10 QDMPs in an organisation.

####  Selected Study Participants

 The selected pharmaceutical company cohort consisted of 31 individuals from the regulatory affairs leadership team (LT) across the company who were responsible for ensuring the regulatory, quality and safety aspects of medicines for the purpose of submission of a new medicine to a regulatory authority; and 3 sub-teams (STs) who were responsible for compiling the data for the leadership committee; 2 of whom focus on medicines’ regulatory aspects and one on their safety.

For the selected regulatory agency, the 47 participants were all assessors who were engaged in reviewing the regulatory dossier prior to the granting of the marketing authorization of a medicine (pre-market assessors) as well as assessors who re-evaluate the marketing authorization status based on new information (post-market assessors). The 28 HTA agency participants were members of the appraisal committee, which recommends whether or not a new medicine should be reimbursed under the national health system. The members were all external experts not directly employed by the agency. As many decisions are made on a daily basis, each organisation selected a key strategic decision-making process of interest (Table 2).

**Table 2:** Decision-Making Processes Assessed With the QoDoS Across the 3 Case Studies

**Study**	**Participant**	**Decision-Making Process Specified for Completing QoDoS**	
		**QoDoS Part 1 (Individual’s Perception of Organization’s Decision-Making)**	**QoDoS Part 2 (Individual’s Perception of Own Decision-Making)**
Pharmaceutical company	LT	LT process to submit a new drug application to a regulatory authority	
	STs: 2 regulatory and one safety	LT’s decision-making to submit a new drug application to a regulatory authority	ST process to present an emerging risk to a regulatory authority
Regulatory authority	Pre-market assessors	Pre-market process to approve or reject a new drug application	
	Post-market assessors	Post-market process to modify (or not) the marketing authorization of a new medicine based on new information	
HTA agency	Appraisal committee members	Committee’s process to recommend/restrict or not to recommend reimbursement of a new medicine, focusing on single technology assessment of pharmaceutical products.	

Abbreviations: QoDoS, Quality of Decision-Making Orientation Scheme; LT, leadership team; ST, Sub-team; HTA, health technology assessment.

###  Study Procedure

 Each study followed the same format to ensure consistency, including introduction to the study through a seminar, followed by completion of the QoDoS. Initial feedback discussions were organized with the cohort leaders in order to review the relevance and clarity of the results as well as their initial perception of the value and benefits of the study methodology. The 3 case studies were completed by February 2018.

###  Data Processing and Analysis

Information was processed into an Excel database for the completed questionnaires where descriptive statistics were used to analyse the dataset. The scores for the individual QoDoS items were codified based on the categorization in Table 1. This was then used to calculate the overall score for each QDMP by taking a median across the relevant QoDoS item scores. Due to confidentiality reasons, only aggregated results are shown here and no data that identify an individual or a specific organisation are reported. No statistical tests were planned or conducted, as this study was designed to be illustrative, with an aim of providing an assessment of the objectives as well as to generate hypotheses for further research.

 The median scores were presented in the form of spider plots and the following traffic light colour coding was used according to the overall QDMP scores, where score <1 = “unfavourable practice” = red; score >1 and <3 = “needs improvement” = yellow; and score >3 = “favourable practice” = green.

## Results

 This evaluation focused on assessing the implementation of the 10 QDMPs using the QoDoS through 3 cross-sectional case studies with a pharmaceutical company, a regulatory authority and an HTA agency. For the purpose of clarity, the results are presented in 3 parts:

- Part I – Pharmaceutical company
- Part II – Regulatory authority
- Part III – HTA agency

###  Characteristics of the Study Participants

The 3 QoDoS case studies were completed by a total of 31 individuals from the 4 teams within pharmaceutical companies, 40 individuals from the regulatory authority and 25 from the HTA agency. The response rate was 100% for the company (31 individuals), 85% for the regulatory authority (25/32 pre-markets assessors; 15/15 post-markets assessors) and 89% for the HTA agency (25/28 individuals) (Table 3).

**Table 3:** Demographic Characteristics of the Study Participants

**Cohort**	**Participant**	**Total Number of Respondents**	**Number of Respondents by Gender**			**Work Experience (Years)**		
			**Male**	**Female**	**Not Specified**	**Median**	**Max**	**Min**
Pharmaceutical company	LT	5	2	3	0	20	36	13
	ST 1 (regulatory)	6	1	3	2	25	37	14
	ST 2 (regulatory)	11	6	3	2	20	32	8
	ST 3 (safety)	9	3	6	0	20	33	11
	Combined company	31	12	15	4	20	37	8
Regulatory authority	Pre-market assessors	25	11	11	3	20	32	2
	Post-market assessors	15	5	9	1	6	37	1
	Combined authority	40	16	20	4	15	37	1
HTA agency	Appraisal committee members	25	15	6	4	24	35	2.5
**Combined (all cohorts)**	**All subjects**	**96**	**43**	**41**	**12**	**21**	**37**	**1**

Abbreviations: LT, leadership team; ST, Sub-team; HTA, health technology assessment.

###  Organisational Feedback Discussions

 Informal feedback discussions were organized with the group leaders from each study to discuss the results. These confirmed the feasibility of the study method as well as initial benefits of the approach, including raising awareness of biases and best practices in decision-making, gaining a basis for discussion of the issues in decision-making and for making recommendations for improving the lowest scoring or least consistent practices. The discussions also helped to uncover the rationale for some differences in responses across sub-groups or when comparing individual and organisational perception and these were incorporated into the relevant results section for the company (part I), regulatory authority (part II) and the HTA agency (part III).

###  Part I – Pharmaceutical Company

####  Assessment of Individual Practices

**Figure 2:**
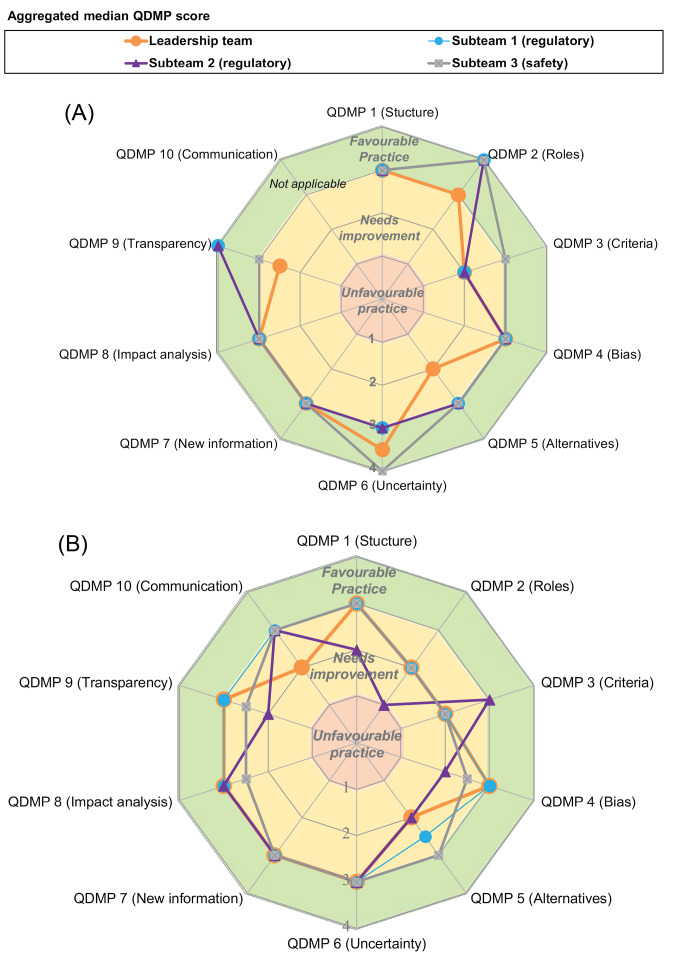
Ten QDMPs for pharmaceutical company LT and the 3 STs (A) Individual’s decision-making (QoDoS Part 2); (B) Perception of Organisation’s Decision-Making (QoDoS Part 1). Abbreviations: QDMPs, Quality Decision-Making Practices; QoDoS, Quality of Decision-Making Orientation Scheme; LT, leadership team; ST, Sub-team.

The individual-level QDMPs of the company participants were generally favourable, namely how the individuals within the 3 STs perceived their decision-making process for presenting an emerging risk to a regulatory authority as well as how the individuals within the LT perceived their process for submitting a new drug application to a regulatory authority (Figure 2A).

 An analysis of the QDMP median scores for the 3 STs revealed that the individual practices were identical and generally favourable for ST1 and ST2, thereby suggesting more consistency across these 2 regulatory STs compared with the safety ST (ST3). One practice that was not favourable for STs 1 and 2 was QDMP 3 (assign values and relative importance to decision criteria), which received a median score of 2. In contrast, the practices for ST3 were generally favourable and differed from STs 1 and 2 regarding QDMP 3 (criteria) and 6 (consider uncertainty) with higher scores for ST3 compared with STs 1 and 2, whereas QDMP 9 (ensure transparency and provide a record trail) received a lower score for the safety group compared with the 2 regulatory STs.

 The main differences between the practices of the 3 STs and the LT were in QDMP 2 (assign clear roles and responsibilities), where the practices of the LT were slightly less favourable, although both generally were good. Similarly, QDMP 5 (examine alternative solutions), where the LT scored in the area of “needing improvement” and QDMP 9 (transparency) was also less favourable for the LT compared with the 3 STs. A potential explanation received from the company during a feedback study session was that this may be due to the fact that the STs have clear remits regarding their roles and responsibilities for assembling the information to the leadership committee, including the decisions made by the individual team members, whereas the LT makes decisions as a group and therefore the group dynamics may result in a perception that there is less transparency around the decision-making process of the LT members, including how alternatives are considered.

The variance in the scores was also explored (Supplementary file 2) and demonstrated that despite some differences in medians, the overlap between the scores was considerable, for example for QDMP 2 (roles) where the median score was between 3 and 4 for the 3 STs, whereas the 25th-75th median range was also between 3 and 4, demonstrating that generally the individual practices are relatively consistent and favourable. On the other hand, some differences in variance exist for practices with the same median, for example QDMP 1 (have a systematic, structured approach to aid decision-making), where the 25th-75th box was in the area of ‘favourable practice’ for ST3, but suggests a potential need for improvement for the LT and ST2. The practice with the most variance across the group was QDMP 3 (criteria), indicating that this practice is not consistently applied across the individuals within the teams.

####  Assessment of Organisational Practices

The individuals from the 3 STs also evaluated their perception of the decision-making of the LT for submitting a new drug application to a regulatory authority, which was compared to the results of the LT assessing their own decision-making for that same decision-making process (Figure 2B). The 3 STs perceived a number of practices of the LT as favourable, namely QDMP 6 (consider uncertainty), QDMP 7 (re-evaluate as new information becomes available), QDMP 8 (perform impact analysis of the decision) and QDMP 10 (effectively communicate the basis of the decision). On the other hand, there were some differences in the median scores between the 3 STs relating to how they each perceived the LT for QDMP 1 (have a systematic, structured approach to aid decision-making), QDMP 2 (assign clear roles and responsibilities), QDMP 3 (assign values and relative importance to decision criteria), QDMP 4 (evaluate both internal and external influences/biases), QDMP 5 (examine alternative solutions) and 9 (ensure transparency and provide a record trail).

The results from the 3 STs were generally similar to those obtained directly from the LT, where one of the main differences was regarding QDMP 10 (effectively communicate the basis of the decision). For QDMP 10, the 3 STs generally agreed that the LT had clear communication practices in place, whereas the LT perceived this practice as “needing improvement.” A potential explanation received during the initial feedback session was that this may be a result of efficient and clear communication of the LT’s decisions by the ST managers to their direct reports in STs 1, 2 and 3; this was highlighted as key to informing the day-to-day activities of the 3 STs. As with the assessment of the individual practices, the perceived incorporation of the 10 QDMPs into the LT’s decision-making was also characterised by considerable variance, for example for QDMP 1 (structure), 2 (roles), 3 (criteria) and 4 (bias) (Supplementary file 2).

###  Part II – Regulatory Authority 

####  Assessment of Individual Practices

**Figure 3:**
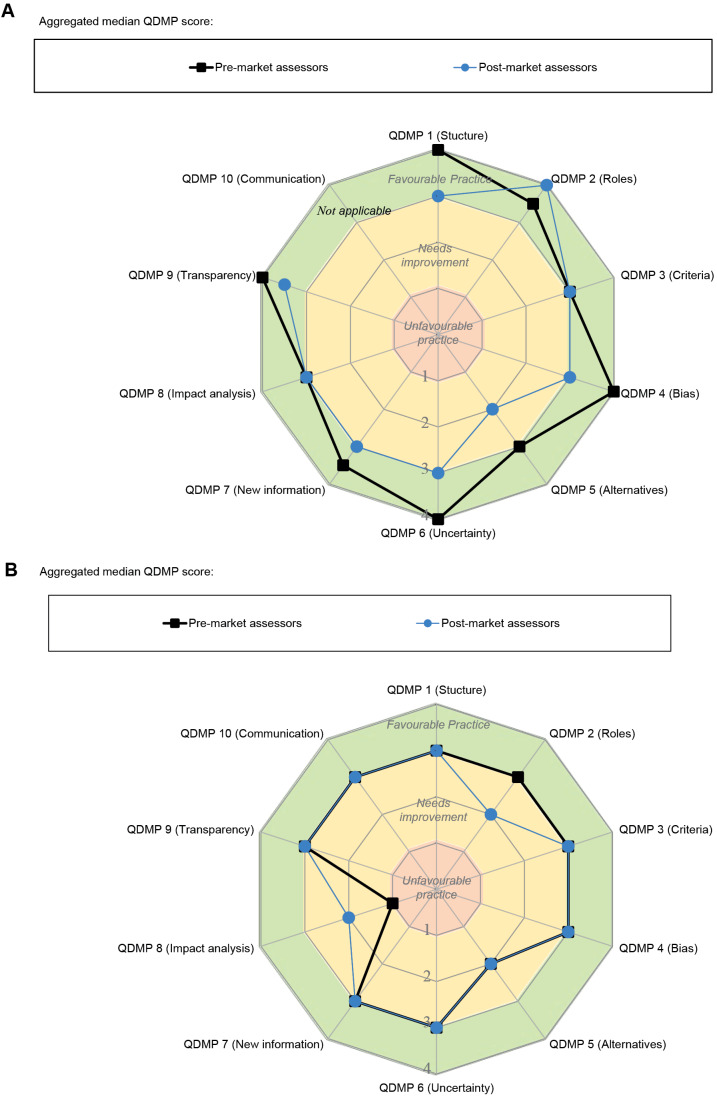
Ten QDMPs for Regulatory Authority Pre- and Post-market Assessors (A) Individual’s Decision-Making (QoDoS Part 2); (B) Perception of Organisation’s Decision-Making (QoDoS Part 1). Abbreviations: QDMPs, Quality Decision-Making Practices; QoDoS, Quality of Decision-Making Orientation Scheme.

An analysis of the decision-making practices across the 40 assessors indicated that both pre-market and post-market assessors perceive their practices as generally “favourable” across all 10 QDMPs (Figure 3A). The only area needing improvement was QDMP 5 (examine alternative solutions) for the post-market assessors. Overall, the practices were consistent between pre- and post-marketing staff; however, some differences in median scores were observed for QDMP 1 (have a systematic, structured approach to aid decision-making) nevertheless the practice was favourable for both. Other differences were observed for QDMP 4 (evaluate both internal and external influences/biases), QDMP 5 (examine alternative solutions) and QDMP 6 (consider uncertainty). Initial discussion with the agency based on their feedback indicated that the rationale for this response was that the decision of the regulator can be binary (to approve or reject) although in other situations there may be variations according to the type of indication, dose, or patient population. In addition, the variance around the median for each of those QDMP (Supplementary file 2) suggests that there are considerable differences around certain practices. For example, despite QDMP 3 (assign values and relative importance to decision criteria) having a median corresponding to ‘favourable practice,’ the variance (25th-75th percentile) was in the area of ‘needing improvement,’ particularly for pre-market assessors, indicating that this practice should be further explored.

####  Assessment of Organisational Practices

The perception of organisational QDMPs by the assessors was also in the area of “favourable practice” and the QDMP scores were similar for pre- and post-market assessors (Figure 3B). However, there was more consistency between responses from pre- and post-market assessors in their perception of their agency (Figure 3B) compared with their own decision-making (Figure 3A). The differences between pre- and post-market assessors (Figure 3B) were for QDMP 2 (assign clear roles and responsibilities), where pre-market responses resulted in “favourable practice” compared with “needing improvement” for post-market staff. In addition, QDMP 8 (perform impact analysis of the decision), was rated by pre-market assessors as “unfavourable practice” and by post-market assessors as “needing improvement.” Based on feedback discussions with the agency, the rationale for this score was that the analysis of impact of decision-making during the regulatory review is not seen as one of the roles of a regulatory agency and therefore may not be applicable.

Finally, there was also considerable variance for a number of the QDMPs, such as QDMP 3 (criteria) and QDMP 5 (alternatives) for pre-market assessors and QDMP 4 (bias) for post-market assesses (Supplementary file 2).

###  Part III – HTA Agency

####  Assessment of Individual and Organisational Practices

**Figure 4:**
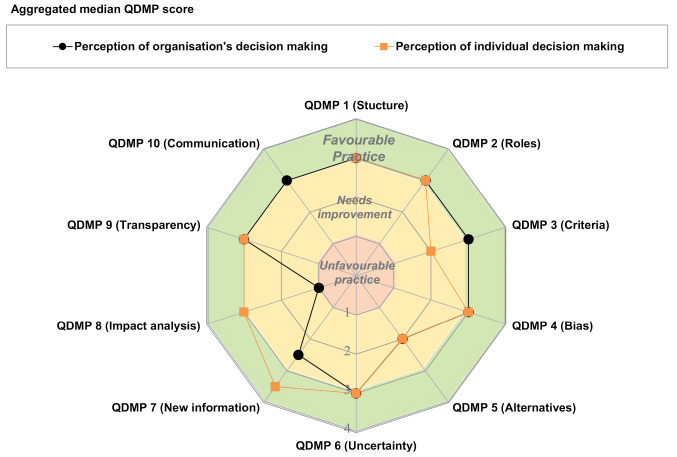
Ten QDMPs for Individuals’ and Organisation’s Decision-Making (QoDoS Parts 2 and 1, Respectively) for HTA Appraisal Committee Members. Abbreviations: QDMPs, Quality Decision-Making Practices; QoDoS, Quality of Decision-Making Orientation Scheme; HTA, health technology assessment.

The combined results for the 25 individuals from the HTA agency appraisal committee showed that the committee members perceived their own decision-making practices as generally “favourable” as well as that of the organisation (Figure 4). The practices that were “unfavourable” or “needing improvement” were QDMP 3 (assign values and relative importance to decision criteria), QDMP 5 (examine alternative solutions) and QDMP 8 (perform impact analysis of the decision), which is similar to the regulatory authority. Minor differences were identified between how the individuals make the decision and how they view the organisation; for example, in QDMP 3, where the assessment of the decision criteria was favourable for the organisation but less so for the individual. The score of the individual QDMP 8 regarding impact analysis suggests an unfavourable practice, which was also the case for the regulatory agency. A number of QDMPs were characterised by considerable variance in the ‘needing improvement’ area, for example QDMP 2 (assign clear roles and responsibilities) and QDMP 7 (re-evaluate as new information becomes available) regarding the individuals’ perception of the organisation’s decision-making (Supplementary file 2).

## Discussion

###  Advancing the Field of Quality Decision-Making

Although there is an increasing use of framework-based quantitative modelling to aid decision-making within the lifecycle of medicines, the various decision-making processes are subjective and often qualitative in nature. Therefore, these should be further explored to identify areas of best practice as well as those that may need improvement to increase process quality, consistency and transparency.^18^ Although, the well-established principles of good decision-making are common sense, they are nevertheless not always common practice. This applies to both how individuals decisions on a daily basis, as well as how key strategic decisions are made across a range of organisations including the pharmaceutical industry.^6^ A recent review of the literature identified general paucity of research in this area, particularly regarding the development and systematic application of techniques for evaluating quality decision-making, such as through the studies undertaken as part of this research.^9^ Indeed this is the first assessment to evaluate quality of decision-making within the different organisations involved in the development, review and reimbursement of medicines.

 Overall, these 3 studies demonstrated the practicality of QoDoS to identify favourable and unfavourable practices as well as to assess their consistency and transparency within as well as across the organisations. Importantly, this study was the first implementation of QoDoS in an in-depth organisational setting and the results confirmed the initial feasibility of the proposed method. All 3 case studies demonstrated generally favourable results across the QDMPs, where overall, all 3 organisations have incorporated the majority of the 10 QDMPs. This may not be surprising, as the organisations that were selected for the 3 studies have established decision-making systems based on their size and multinational status (for the company) and maturity level (in case of agencies).

###  Application of QoDoS in the Development and Review of Medicines

 The 3 case studies differed in terms of cohort type and decision point in order to demonstrate the different ways in which QoDoS can be implemented. The pharmaceutical company study was used to assess the decision-making across 4 groups (3 STs and one LT), where the QoDoS questions relating to the perception of the organisation were used to assess the LT based on the LT’s perception as well as that of the 3 STs. In this case, the decision-making of the 4 teams was generally very similar, demonstrating good incorporation of QDMPs across the organisation.

 Second, the regulatory authority study was used to demonstrate differences in individual practices and perception of the organisation by 2 different reviewer groups (pre- and post-market approval). In this case, the QoDoS responses demonstrated that the perception of the organisation is relatively consistent for the 2 groups and certain differences were identified in how the individuals make decisions, which may be due to the different processes in place for the pre- and post-marketing activity.

 Lastly, the HTA case study involved one group, the appraisal committee, where the individuals assessed themselves and their organisation for the same decision point, where the results demonstrated general consistency.

 Despite the fact that the scope and the decision-making processes within each organisation are different, it could be argued that each organisation should be implementing the same practices to promote quality and consistency. Although, in general, the outcomes of the 3 cohorts were very positive in demonstrating alignment in terms of the incorporation of the QDMPs, the QoDoS demonstrated the need for improvement across a number of practices, where some similarities were identified, such as the need for better assignment of values and relative importance of decision criteria (QDMP 3) as well as the evaluation of alternatives (QDMP 5) for the 3 organisations and the need for impact analysis by the individuals within the regulatory and the HTA agency appraisal committee (QDMP 8).

Interestingly, both QDMP 3 and QDMP 8 were seen as generally not incorporated into company and agency frameworks during medicines regulatory submission, review, and HTA processes.^19^ As these practices were nevertheless seen as relevant by the respondents, QDMP 3 should be addressed through the incorporation of more formal frameworks, such as a benefit-risk framework during regulatory decision-making,^20^ and through having standardized evidence criteria for HTA.^21^

Although both the regulatory agency and the HTA agency felt that impact analysis may be out of scope for the agency review and appraisal, this may be due to the interpretation of what impact analysis entails and in fact, may already be an intrinsic part of the decision-making process. Indeed, impact analysis (QDMP 8); such as an analysis of the impact on patients, society and other agencies, could still be considered as the remit of agencies. The practice could be incorporated within frameworks in 2 ways; first, through an assessment of linked decisions such as relevant precedents, including decisions previously made by the organisation or other relevant stakeholders.^1^ Second, such an assessment should focus on the impact of this decision on present and future processes and should address relevant stakeholders including patients.^22^ For example, a pre-marketing authorization regulatory impact analysis would enable assessment of how similar medicines were reviewed (including outcomes) within their own jurisdiction and by other regulators; in addition, the analysis would facilitate understanding of the impact of the decision on other processes, such as HTA, as well as the ultimate effect of the decision (including the approved label) on patients.

 Another key finding was the general variance (25th-75th percentile) around the responses obtained from the 3 groups for the incorporation of the 10 QDMPs. It is important to note that variation is not perceived as a shortcoming, as QoDoS assesses a process that is subjective in nature and aims to capture differences in perceptions that can then be explored through feedback discussions. Differences in scores may be a result of a mixture of factors: inconsistencies in individual practices and differences in the perception of the organisation due to poor transparency and documentation of the practices or different experiences within the organisation.

###  Next Steps for Improving the Practices of Individuals and Organisations

 The initial discussions with the cohort leaders gave an early indication of the benefits of the implementation of the QoDoS; for example, having a basis to identify issues in decision-making in order to improve practices. The 3 organisations should now determine the best way forward to change any decision-making practices that have been shown to be inconsistent or to need improvement.

It would also be of interest to initiate similar studies with additional teams and groups from each of the 3 organisations to assess consistency of QDMPs eg, discovery, pre-clinical and clinical drug development in companies; as well as other committees, reviewers and project managers within agencies. It would also be of value to involve other organisations, including smaller companies or agencies from emerging markets with less established systems, to determine how QDMPs are built into those organisations, compared with larger companies and agencies.^23^ This could in turn help improve the productivity of companies and ensure agencies are not only undertaking a good-quality review or appraisal but are also making sure their decisions are consistent and transparent.

###  Limitations

 Only one company, regulatory authority and HTA agency were selected to demonstrate the practicality of QoDoS for identifying the incorporation of the 10 QDMPs and generally to demonstrate the feasibility of the method. This sampling was considered nevertheless appropriate as the aim was to produce illustrative case studies for testing the practical application of the methodology, as opposed to generating aggregated trends or extrapolating the results to other organisations. Furthermore, the feedback discussions with the study participants were so far limited and it would be of interest to determine what changes were implemented based on the results and what impact they had on the decision-making practices going forward if a QoDoS study could be repeated in the future. Finally, other appropriate tools may exist to undertake such assessments, particularly if they were not published.

## Conclusion

QoDoS can be applied as a diagnostic instrument within organisations for the routine assessment of quality of decision-making. Importantly, the data obtained from QoDoS studies could provide a basis for internal dialogue (within an organisation) and to ultimately address the least favourable practices by incorporating them more effectively into decision-making frameworks. The publication of such results may also help minimise reputational risk of an organisation, by demonstrating that best practices are in place (or improvements are being made) and therefore to increase trust in the process. Such trust is key both from patients to understand the decision-making process, as well as from other stakeholders particularly in the case of agencies aligning their decision-making processes.^5,6^

 In addition, the routine assessment of the QDMPs has the ability to measure change over time in order to determine the impact of any improvement initiatives. The ongoing use of quality frameworks for making decisions will also reduce uncertainty around decision-making and might result in more predictable and favourable outcomes. Finally, QoDoS can be used to externally benchmark an organisation’s decision-making practices with other organisations. This in turn could be used to promote best practices as well as to build alignment into key strategic decisions made by organisations during medicines development, regulatory review and reimbursement.

## Acknowledgements

 The authors wish to thank the individuals from the pharmaceutical company, the regulatory authority and the health technology assessment agency who took part in the case studies and facilitated timely completion of the work.

## Ethical issues

 The study protocol received approval from the University of Hertfordshire Institutional Ethics Committee. Since the study participants were neither National Health Service patients or staff, it did not require the Local Ethics Committee approval. The eligible participants received a copy of the “Participant Information Sheet” describing the purpose of the study, reasons for their participation and explaining that only aggregated results will be reported, before they decide to agree or decline to take part in the study. Therefore, their agreement to participate in the study constituted consent.

## Competing interests

 Authors declare that they have no competing interests.

## Authors’ contributions

 MB designed the study, analyzed the data, and wrote the manuscript. NM designed the study and wrote the manuscript. SW designed the study and wrote the manuscript. SS designed the study and wrote the manuscript.

## Authors’ affiliations

^1^Department of Clinical and Pharmaceutical Sciences, School of Life & Medical Sciences, University of Hertfordshire, Hatfield, UK. ^2^Centre for Innovation in Regulatory Science (CIRS), London, UK.

## Supplementary files


Supplementary file 1. Background Information on the Development and Validation of the QDMPs and the QoDoS.

Supplementary file 2. Variance in the QDMP Scores.
