# Supplementary material for: A Process for Evaluating Quality Decision-Making Practices During the Development, Review and Reimbursement of Medicines
Source: Int J Health Policy Manag. 2020 Jun 20;11(2):128–37. doi: 10.34172/ijhpm.2020.86 (PMC9278608; doi:10.34172/ijhpm.2020.86)
Supplement: Supplementary file 1 — Background Information on the Development and Validation of the QDMPs and the QoDoS. [file ijhpm-11-128-s001.pdf]

Supplementary file 1. Background Information on the Development and Validation of the QDMPs) and the Quality of Decision-Making Orientation Scheme (QoDoS)

In the absence of an appropriate instrument for assessing quality decision-making practices (QDMPs) during medicines development,[1] a study was initiated to develop a standardised approach to QDMPs and validate an instrument, the Quality of Decision-Making Orientation Scheme (QoDoS). This was carried out in 4 phases outlined below.

Phase 1: Qualitative study The QoDoS items were generated from face-to-face semi-structured interviews with 29 key opinion leaders from the pharmaceutical industry, contract research organisations and regulatory agencies.[2] Content validity was established using an expert panel to confirm that the emphasis and the focus of the QoDoS is fit-for-purpose (ie, being robust, relevant and sound). A major outcome of this study has also been the identification of the 10 QDMPs that underpin a quality process. These were considered as relevant by pharmaceutical companies, regulatory agencies and HTA agencies.[3,4] The QoDoS items can be grouped according to these 10 practices, and consequently the incorporation of the practices into company and agency processes can be assessed with the instrument.

Phase 2: Psychometric analysis and quantitative stage Psychometric evaluations including factor analysis, reliability and construct validation were also performed.[5,6] This study resulted in a 47-item QoDoS instrument organised into 4 sections namely, organisational decision-making approaches, organisational decision-making culture, individual decision-making competencies and individual decision-making style.

Phase 3: Practicality and applicability Finally, the practicality and applicability of QoDoS to evaluate quality decision-making was assessed through a study with 76 participants from the pharmaceutical industry and regulatory agencies. The findings demonstrated that the QoDoS has the ability to identify differences in decision making between individuals and their organization as well as differences between companies and agencies.[7]

Phase 4: Organisational case studies with QoDoS Following its validation, QoDoS is currently being implemented across participants from specific teams, committees or departments from across companies, regulatory and HTA agencies. Such studies are being be used to determine the factors that influence decision making within organisations, including favourable practices and those that might require

improvement, as well as to identify common themes in quality decision making across the different organisations.

1. Bujar M, McAuslane N, Walker SR and Salek S. Evaluating Quality of Decision-Making Processes in Medicines' Development, Regulatory Review, and Health Technology Assessment: A Systematic Review of the Literature. *Front Pharmacol.* 2017; 8:189.
2. Donelan R, Walker S, Salek S. Factors influencing quality decision-making: regulatory and pharmaceutical industry perspectives. *Pharmacoepidemiol Drug Saf.* 2015; 24(3) pp. 319–328.
3. Bujar M, McAuslane N, Salek S, Walker S. Quality of regulatory decision-making practices: issues facing companies and agencies. Ther Inn Reg Sci. 2016b;DOI: 10.1177/2168479016628573.
4. Bujar M, McAuslane N, Walker SR and Salek S. Quality Decision Making in Health Technology Assessment: Issues Facing Companies and Agencies. *Ther Inn Reg Sci.* 2019; DOI: 10.1177/2168479019833660.
5. Donelan R, Walker S, Salek S. The development and validation of a generic instrument, QoDoS, for assessing the quality of decision making. *Front Pharmacol.* 2016; Jul 13;7:180.
6. Bujar M, McAuslane N, Walker S, Salek S. The Reliability and Relevance of a Quality of Decision Making Instrument, Quality of Decision-Making Orientation Scheme (QoDoS), for Use During the Lifecycle of Medicines. *Front Pharmacol.* 2019;10:17. doi:10.3389/fphar.2019.00017
7. Bujar, M., Donelan, R., McAuslane, N., Walker, S., Salek, S. Assessing the quality of decision making in the development and regulatory review of medicines: identifying biases and best practices. *Ther Innov Reg Sci.* 2016; 51, 250–256.
