# Supplementary material for: A Process for Evaluating Quality Decision-Making Practices During the Development, Review and Reimbursement of Medicines
Source: Int J Health Policy Manag. 2020 Jun 20;11(2):128–37. doi: 10.34172/ijhpm.2020.86 (PMC9278608; doi:10.34172/ijhpm.2020.86)
Supplement: Supplementary file 2 — Variance in the QDMP Scores. [file ijhpm-11-128-s002.pdf]

Supplementary file 2. Variance in the Quality Decision-Making Practice (QDMP) scores

Pharmaceutical company LT and the 3 STs: individual's decision making (QoDoS Part 2)

		Score - Percentile values			
**Quality decision-making practice (QDMP)**	**Cohort**	**25th**	**50th**	**75th**	**Difference 25-75th**
**QDMP 1 (Structure)**	**LT**	2	3	3	1
	**ST1**	3	3	3	0
	**ST2**	2	3	3	1
	**ST3**	3	3	4	1
**QDMP 2 (Roles)**	**LT**	3	3	4	1
	**ST1**	3.25	4	4	0.75
	**ST2**	3	4	4	1
	**ST3**	3	4	4	1
**QDMP 3 (Criteria)**	**LT**	1	2	3	2
	**ST1**	1	2	3	2
	**ST2**	1	2	3	2
	**ST3**	1	3	3	2
**QDMP 4 (Bias)**	**LT**	2.25	3	3	0.75
	**ST1**	3	3	4	1
	**ST2**	2	3	3	1
	**ST3**	3	3	4	1
**QDMP 5 (Alternatives)**	**LT**	2	2	3	1
	**ST1**	3	3	3.75	0.75
	**ST2**	2	3	3	1
	**ST3**	2	3	4	2
**QDMP 6 (Uncertainty)**	**LT**	2.25	3.5	4	1.75
	**ST1**	3	3	4	1
	**ST2**	3	3	4	1
	**ST3**	3	4	4	1
**QDMP 7 (New information)**	**LT**	3	3	3	0
	**ST1**	2	3	3	1
	**ST2**	3	3	3.75	0.75
	**ST3**	3	3	4	1
**QDMP 8 (Impact)**	**LT**	3	3	4	1
	**ST1**	3	3	4	1
	**ST2**	3	3	3	0
	**ST3**	3	3	4	1
**QDMP 9 (Transparency)**	**LT**	2	2.5	3	1
	**ST1**	3	4	4	1
	**ST2**	3	4	4	1
	**ST3**	3	3	4	1

Pharmaceutical company LT and the 3 STs: perception of organisation's decision making

		Score - Percentile values			
**Quality decision-making practice (QDMP)**	**Cohort**	**25th**	**50th**	**75th**	**Difference 25-75th**
**QDMP 1 (Structure)**	**LT**	1	3	3	2
	**ST1**	2	3	3	1

	**ST2**	1	2	3	2
	**ST3**	2	3	3	1
**QDMP 2 (Roles)**	**LT**	1	2	2.75	1.75
	**ST1**	0.5	2	3	2.5
	**ST2**	1	1	2	1
	**ST3**	1.75	2	3	1.25
**QDMP 3 (Criteria)**	**LT**	1.25	2	4	2.75
	**ST1**	1.75	2	3	1.25
	**ST2**	1.25	3	3	1.75
	**ST3**	1	2	3	2
**QDMP 4 (Bias)**	**LT**	1	3	3	2
	**ST1**	1.5	3	3	1.5
	**ST2**	1	2	3	2
	**ST3**	2	2.5	3	1
**QDMP 5 (Alternatives)**	**LT**	1.25	2	3	1.75
	**ST1**	2	2.5	3	1
	**ST2**	1.5	2	2	0.5
	**ST3**	2.25	3	4	1.75
**QDMP 6 (Uncertainty)**	**LT**	3	3	3	0
	**ST1**	2.75	3	3	0.25
	**ST2**	2	3	4	2
	**ST3**	2	3	4	2
**QDMP 7 (New information)**	**LT**	2.25	3	3	0.75
	**ST1**	2.5	3	3	0.5
	**ST2**	3	3	3	0
	**ST3**	3	3	3.75	0.75
**QDMP 8 (Impact)**	**LT**	3	3	3	0
	**ST1**	3	3	3.75	0.75
	**ST2**	2	3	4	2
	**ST3**	1.75	2.5	4	2.25
**QDMP 9 (Transparency)**	**LT**	3	3	3	0
	**ST1**	2	3	3.5	1.5
	**ST2**	1	2	3	2
	**ST3**	1.75	2.5	3	1.25
**QDMP 10 (Communication)**	**LT**	2	2	3	1
	**ST1**	1.5	3	3	1.5
	**ST2**	1.5	3	3	1.5
	**ST3**	2	3	3	1

Regulatory authority pre- and post-market assessors: individual's decision-making

		**Score - Percentile values**			
**Quality decision-making practice (QDMP)**	**Cohort**	**25th**	**50th**	**75th**	**Difference 25-75th**
**QDMP 1 (Structure)**	**Pre-market**	3	4	4	1
	**Post-market**	2	3	4	2
**QDMP 2 (Roles)**	**Pre-market**	3	3.5	4	1
	**Post-market**	4	4	4	0

**QDMP 3 (Criteria)**	**Pre-market**	1	3	4	3
	**Post-market**	2	3	3	1
**QDMP 4 (Bias)**	**Pre-market**	3	4	4	1
	**Post-market**	3	3	4	1
**QDMP 5 (Alternatives)**	**Pre-market**	1	3	3	2
	**Post-market**	1.5	2	3	1.5
**QDMP 6 (Uncertainty)**	**Pre-market**	3	4	4	1
	**Post-market**	3	3	4	1
**QDMP 7 (New information)**	**Pre-market**	3	3.5	4	1
	**Post-market**	3	3	3	0
**QDMP 8 (Impact)**	**Pre-market**	3	3	4	1
	**Post-market**	2	3	3	1
**QDMP 9 (Transparency)**	**Pre-market**	3	4	4	1
	**Post-market**	3	3.5	4	1

Regulatory authority pre- and post-market assessors: perception of organisation's decision making

		**Score - Percentile values**			
**Quality decision-making practice (QDMP)**	**Cohort**	**25th**	**50th**	**75th**	**Difference 25-75th**
**QDMP 1 (Structure)**	**Pre-market**	2	3	4	2
	**Post-market**	2	3	3	1
**QDMP 2 (Roles)**	**Pre-market**	2	3	3	1
	**Post-market**	2	2	3	1
**QDMP 3 (Criteria)**	**Pre-market**	1.25	3	4	2.75
	**Post-market**	1.5	3	3	1.5
**QDMP 4 (Bias)**	**Pre-market**	3	3	4	1
	**Post-market**	1.25	3	4	2.75
**QDMP 5 (Alternatives)**	**Pre-market**	1	2	3.5	2.5
	**Post-market**	1	2	3	2
**QDMP 6 (Uncertainty)**	**Pre-market**	3	3	4	1
	**Post-market**	3	3	4	1
**QDMP 7 (New information)**	**Pre-market**	2	3	4	2
	**Post-market**	2	3	3	1
**QDMP 8 (Impact)**	**Pre-market**	1	1	2	1
	**Post-market**	1	2	3	2

**QDMP 9 (Transparency)**	**Pre-market**	3	3	4	1
	**Post-market**	2	3	3	1
**QDMP 10 (Communication)**	**Pre-market**	2	3	4	
	**Post-market**	1.5	3	3	

HTA appraisal committee members: individual's decision making

	**Score - Percentile values**			
**Quality decision-making practice (QDMP)**	**25th**	**50th**	**75th**	**Difference 25-75th**
**QDMP 1 (Structure)**	2	3	4	2
**QDMP 2 (Roles)**	2	3	4	2
**QDMP 3 (Criteria)**	1	2	3	2
**QDMP 4 (Bias)**	3	3	3	0
**QDMP 5 (Alternatives)**	1	2	3	2
**QDMP 6 (Uncertainty)**	3	3	4	1
**QDMP 7 (New information)**	3	3.5	4	1
**QDMP 8 (Impact)**	3	3	4	1
**QDMP 9 (Transparency)**	2	3	3	1

HTA appraisal committee members: perception of organisation's decision making

	**Score - Percentile values**			
**Quality decision-making practice (QDMP)**	**25th**	**50th**	**75th**	**Difference 25-75th**
**QDMP 1 (Structure)**	2	3	4	2
**QDMP 2 (Roles)**	1	3	3	2
**QDMP 3 (Criteria)**	1.75	3	4	2.25
**QDMP 4 (Bias)**	3	3	4	1
**QDMP 5 (Alternatives)**	1	2	3	2
**QDMP 6 (Uncertainty)**	3	3	4	1
**QDMP 7 (New information)**	1	2.5	3	2
**QDMP 8 (Impact)**	1	1	3	2
**QDMP 9 (Transparency)**	3	3	4	1
**QDMP 10 (Communication)**	3	3	4	1
